# Pathologies Associated with Serum IgG4 Elevation

**DOI:** 10.1155/2012/602809

**Published:** 2012-08-26

**Authors:** Mikael Ebbo, Aurélie Grados, Emmanuelle Bernit, Frederic Vély, José Boucraut, Jean-Robert Harlé, Laurent Daniel, Nicolas Schleinitz

**Affiliations:** ^1^Université de la Méditerranée Aix-Marseille II, France; ^2^Service de Médecine Interne, Hôpital de la Conception, Assistance Publique-Hôpitaux de Marseille, 13385 Marseille Cedex 5, France; ^3^Laboratoire d'Immunologie, Hôpital de la Conception, Assistance Publique-Hôpitaux de Marseille, 13385 Marseille Cedex 5, France; ^4^Centre d'Immunologie de Marseille-Luminy, Université de la Méditerranée, Case 906, Campus de Luminy, 13288 Marseille Cedex 09, France; ^5^Service d'Anatomie Pathologique et de Neuropathologie, Hôpital de la Timone, Assistance Publique—Hôpitaux de Marseille, 13385 Marseille Cedex 5, France

## Abstract

*Statement of Purpose*. IgG4-related disease (IgG4-RD) is usually associated to an increase of serum IgG4 levels. However other conditions have also been associated to high serum IgG4 levels. *Methods*. All IgG subclasses analyses performed in our hospital over a one-year period were analyzed. When IgG4 level were over 1.35 g/L, the patient's clinical observation was analyzed and both final diagnosis and reason leading to IgG subclasses analysis were recorded. Only polyclonal increases of IgG4 were considered. *Summary of the Results*. On 646 IgG subclass analysis performed, 59 patients had serum IgG4 over 1.35 g/L. The final diagnosis associated to serum IgG4 increase was very variable. Most patients (25%) presented with repeated infections, 13.5% with autoimmune diseases, and 10% with IgG4-RD. Other patients presented with cancer, primary immune deficiencies, idiopathic interstitial lung disease, cystic fibrosis, histiocytosis, or systemic vasculitis and 13.5% presented with various pathologies or no diagnosis. Mean IgG4 levels and IgG4/IgG ratio were higher in IgG4-RD than in other pathologies associated to elevated IgG4 levels. *Conclusions*. Our study confirms that elevation of serum IgG4 is not specific to IgG4-RD. Before retaining IgG4-RD diagnosis in cases of serum IgG4 above 1.35 g/L, several other pathological conditions should be excluded.

## 1. Introduction

Immunoglobulin G4 (IgG4) represents the less abundant of the four IgG subclasses in human serum accounting for 3 to 6% of the total IgG [1].

IgG4 has been associated with several pathological conditions. Most of these associations suggest a protective effect of IgG4, such as in allergen-specific immunotherapy [2] and protection from inflammatory manifestations during parasitosis [3]. In few situations, IgG4 is associated with a direct pathogenic effect, such as in pemphigus. During this blistering dermatosis, antidesmosome autoantibodies belong to the IgG4 subclass [4]. However, total IgG4 serum levels are not raised in these conditions.

In 2001, Hamano et al. report a quantitative serum IgG4 elevation during sclerosing (or “autoimmune”) pancreatitis above the cutoff value of 135 mg/dL in 95% of patients with autoimmune pancreatitis [1]. This entity was first described in 1961 by Sarles and colleagues in patients with lymphoplasmacytic infiltrate and fibrosis of the pancreas associated to polyclonal hypergammaglobulinemia [5]. Polyclonal hypergammaglobulinemia raised initially the issue of the possible auto-immune nature of the disease, but this hypothesis has not been confirmed to date. Indeed, no specific autoantibody has been associated with auto-immune pancreatitis. Serum IgG4 elevation becomes from this date a biological marker of sclerosing (or “auto-immune”) pancreatitis. Other fibroinflammatory organ involvements with similar histopathological characteristics have since been reported, associated or not with pancreatic involvement, in a context of serum IgG4 elevation [6], leading to the concept of an IgG4-related disease [7]. To date, serum IgG4 elevation is considered as a diagnosis criteria for IgG4-related disease [8–10]. However, serum IgG4 elevation is not necessary for the diagnosis, as proposed by the diagnosis criteria, and IgG4 elevation is not specific of the disease [11]. Serum IgG4 elevation has also been reported in various pathological situations: multicentric Castleman's disease [12], Wegener's granulomatosis [13], Churg-Strauss syndrome [14], or pancreatic adenocarcinoma [15]. 

Few works have systematically studied diagnosis associated with a serum IgG4 elevation [11, 16, 17]. In order to better know diagnosis associated with this biological situation, we studied retrospectively all IgG4 subclass measurements achieved during a one-year period at the University Hospital of Marseille, France.

## 2. Materials and Methods

All results for IgG subclasses evaluation performed from January 1^st^, 2009 to December 31th, 2009 at the laboratory of Immunology of our University Hospital of Marseille, France, were analyzed. Serum total IgG and IgG subclasses (1 to 4) levels were measured by immunonephelometry (Siemens Nephelometer Analyser II) with reagents from Siemens (NAS IgG1, NAS IgG2, N latex IgG3, and N Latex IgG4). All results with serum IgG4 polyclonal increase above the cutoff value of 1.35 g/L were considered for the study. 

The patients' medical records were retrospectively analyzed and demographic, clinical, paraclinical, and evolutive characteristics were recorded. The reason leading the physician to the prescription of IgG subclass evaluation was also recorded. Patients were classified according to the disease associated to IgG4 elevation. Mean values in different groups were compared using the Mann-Whitney test. Results were considered significant for *P* < 0.05. Figures and statistics were realized using GraphPad Prism v. 4.0.

## 3. Results

### 3.1. Patients' Characteristics

A total of 646 IgG subclass analyses were recorded at the laboratory of Immunology during the one-year period. An IgG4 level above 1.35 g/L was found on 75 samples that corresponded to 60 different patients (some of them took 2 or more samples during the year). Among these 60 patients, data could be collected for 59 patients. 

Among the 59 patients analyzed, 30 were men and 29 were women (*sex ratio *1/1). Mean age at IgG subclass measurement was 47.2 years (range: 4–85 years). Mean serum IgG level was 18.50 g/L (range: 7.38–40.4 g/L). Thirty-eight patients (64%) presented an elevated serum IgG level (>14 g/L). By definition, a serum IgG4 level above 1.35 g/L was present in all 59 patients. Mean serum IgG4 level was 4.35 g/L (range: 1.37–20.6 g/L).

The reasons leading the physicians to perform IgG subclass analysis are presented in Table 1. Suspicion of immune deficiency (with or without hypogammaglobulinemia) was the first indication for IgG subclass measurement in 21 patients (35.6%). Polyclonal hypergammaglobulinemia was the reason leading to serum IgG subclass measurement in 15 patients (25.4%), IgG4-related disease (IgG4-RD) suspicion in 14 patients (23.7%) with one or more compatible organ involvement. In 9 patients (15.3%), the reason was unclear and classified as other. 

### 3.2. Diagnosis Associated with Serum IgG4 over 1.35 g/L

Final diagnosis in patients who presented with elevated levels of serum IgG4 was divided into different categories presented in Table 2. Most represented categories were repeated infections with 15 patients (25.4%). Site of infections (pneumonia, sinusitis, skin and soft tissues infections, osteitis, and pericarditis) and microorganisms implicated (community-acquired bacteria, *Staphylococcus* sp., herpes virus group (HPV, HSV, and EBV), *Nocardia*, *Toxoplasma gondii*, *Enterobius vermicularis*) were variable in this group. In 8 patients (13.6%), final diagnosis was an autoimmune disease: systemic lupus erythematosus (SLE) in 4 patients (with secondary antiphospholipid syndrome in 2), Sjögren Syndrome (SS) in 2 patients, Biermer disease in 1 patient, and systemic sclerosis in 1 patient. An IgG4-related disease (IgG4-RD) was the final diagnosis in 6 patients (10.1%). Histological documentation was available in all cases of IgG4-RD with characteristic histopathological features with lymphocytic and plasmacytic polyclonal inflammatory infiltrate (with predominant IgG4 positive plasma cells when immunohistological study where available, *n* = 5) and fibrosis. Organ involvements included sclerosing pancreatitis in 4 patients, tubulointerstitial nephritis in 5 patients, polyadenopathy in 5 patients, sialadenitis in 3 patients, sclerosing cholangitis in 2 patients, dacryoadenitis in 1 patient, hypophysitis in one patient, and inflammatory pseudotumors (hepatic in one case, orbital in another case). Four of these patients with IgG4-RD were under treatment (corticosteroids and/or immunosuppressive therapy) at the time of IgG subclass measurement. In 5 patients (8.5%), an IgG4-RD was considered as possible. These patients presented with one or more compatible organ involvements but without histopathological documentation. Sclerosing cholangitis was presented in 4 cases (with renal involvement in one of these patients), sclerosing pancreatitis in the other case. In 4 patients (6.8%) the final diagnosis was a cancer: ampullary carcinoma, angioimmunoblastic T-cell lymphoma, pancreatic carcinoma, and bronchopulmonary carcinoma. Primary immune deficiency (PID) was the final diagnosis in 4 cases (6.8%): Wiskott-Aldrich syndrome, chronic granulomatous disease, common variable immunodeficiency, and complex humoral immune deficiency. Hypogammaglobulinemia on blood electrophoresis was found in 3 of these patients. Idiopathic interstitial pneumonitis was the final diagnosis in 3 patients (5%) (with pulmonary fibrosis in 2 cases). Cystic fibrosis, Erdheim-Chester disease and vasculitis (hepatitis C-virus-associated type-II-mixed cryoglobulinemia in one case and microscopic polyangiitis in another case) were, respectively, diagnosed in 2 patients (3.4%). In 8 patients (13.6%), no final diagnosis could be retained. 

Apart from the diagnosis category considered, allergic and atopic manifestations (allergic rhinoconjunctivitis, nasal polyps and allergic chronic rhinosinusitis, asthma and bronchial hyperreactivity, urticarial skin lesions, and angioedema, hypereosinophilia and/or IgE elevation) were found in the record of only 10 patients (16.9%).

### 3.3. Serum IgG4 Levels Are Significantly Higher in IgG4-RD Than in Other Pathologies

**Figure 1:**
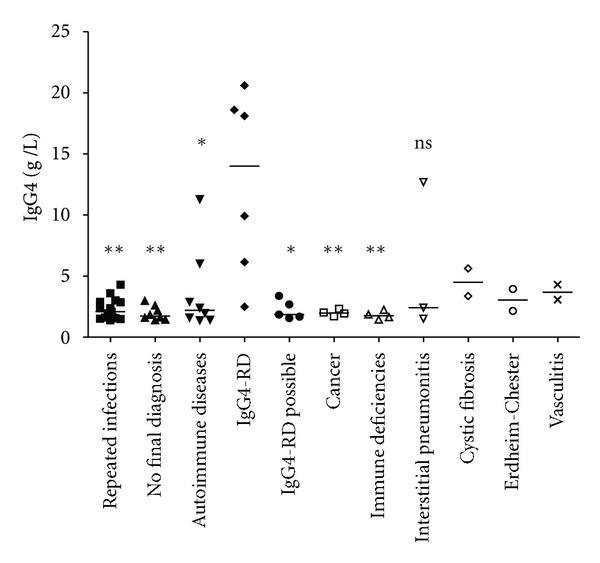
Serum IgG4 levels in different final diagnostic categories of patients with serum IgG4 elevation. IgG4-RD = IgG4-related disease. Horizontal bars represent median values observed in each group. Results obtained in each group were compared to results obtained in IgG4-RD group (Mann-Whitney test): **P < 0.005; *P < 0.05; ns: not significant.

Serum IgG4 levels found in patients of each diagnosis category are presented in Figure 1. Serum IgG4 elevation observed in IgG4-RD group was significantly more important than in the groups “repeated infections” (*P* = 0.0021), “auto-immune diseases” (*P* = 0.0127), “absence of diagnosis” (*P* = 0.0027), “possible IgG4-RD” (*P* = 0.0173), “cancer” (*P* = 0.0095), and “primary immune deficiency” (*P* = 0.0095). Difference with the group “idiopathic interstitial pneumonitis” was not statistically significant (*P* = 0.1667). Statistical analysis with the groups “cystic fibrosis,” “Erdheim-Chester disease,” and “vasculitis” was not possible because of the too small size of these groups (*n* < 3).

**Figure 2:**
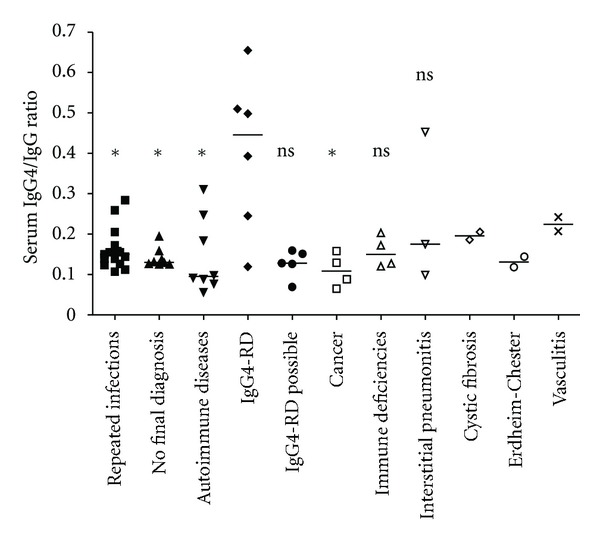
Serum IgG4/serum IgG ratios in different final diagnostic categories of patients with serum IgG4 elevation. IgG4-RD = IgG4-related disease. Horizontal bars represent median values observed in each group. Results obtained in each group were compared to results obtained in IgG4-RD group (Mann-Whitney test): *P < 0.05; ns: not significant.

An analysis of the serum IgG4/serum IgG *ratio* was performed. Serum IgG4/serum IgG *ratios* observed in patients of each diagnosis category are presented in Figure 2. Elevation of serum IgG4/serum IgG *ratio* observed in IgG4-RD group was significantly more important than in the groups “repeated infections” (*P* = 0.0217), “auto-immune diseases” (*P* = 0.0127), “absence of diagnosis” (*P* = 0.0426), and “cancer” (*P* = 0.0381). Differences with the groups “possible IgG4-RD” (*P* = 0.0519), “primary immune deficiency” (*P* = 0.1143), and “idiopathic interstitial pneumonitis” (*P* = 0.2619) were not statistically significant. Statistical analysis with the groups “cystic fibrosis,” “Erdheim-Chester disease,” and “vasculitis” was not possible because of the too small size of these groups (*n* < 3).

## 4. Discussion

Serum IgG4 elevation above 1.35 g/L has been shown to be a predictive marker of type 1 AIP [1] and IgG4-related sclerosing cholangitis [39]. Usefulness of serum IgG4 in diagnosis of AIP has been evaluated for sensibility and specificity [40] and the cutoff value of 1.35 g/L (135 mg/dL) proposed by Hamano in 2001 is widely accepted in literature. To better distinguish AIP from pancreatic cancer more elevated IgG4 cutoff values (140 mg/dL [21] or 280 mg/dL [18]) have been proposed.

In IgG4-RD, the Japan G4 team has retained the elevation of serum IgG4 above 1.35 g/L as an individual diagnosis criteria, [10]. In fact, according to these criteria the presence of a clinical or radiological organ involvement with IgG4 above 1.35 g/L is sufficient to retain the diagnosis of possible IgG4-RD [10]. 

However, IgG4 elevation has been reported in several well-characterized pathologies as pancreatic carcinoma (10% for a cut-off of 140 mg/dL, 1% for a cut-off of 280 mg/dL [41]), allergic diseases [2], parasitic infections [3], and systemic diseases [11]. The different pathological situations associated to serum IgG4 elevation in literature are reported in Table 3.

Thus, exclusion criteria, including pathologies other than IgG4-RD, should be added to the diagnosis criteria as proposed by recent CDC criteria for IgG4-RD [10]. To better characterize pathologies associated with serum IgG4 above 1.35 g/L which could be discussed in the differential diagnosis of IgG4-RD, we retrospectively studied all IgG subclass measurements performed over a one-year period at our university hospital. Recruitment was largely including pediatric and adult patients without restricted specialized medical field. 

Only 10% of 59 patients with elevated serum IgG4 (>1.35 g/L) were diagnosed with IgG4-RD. Other diagnoses associated were infections, auto-immune diseases, cancers, primary immune deficiencies, idiopathic interstitial pneumonitis, or vasculitis. Only patients with IgG subclass evaluation and IgG4 above 1.35 g/L were included in this retrospective study without any control group. Thus, the specificity or the sensitivity of serum IgG4 elevation for diagnosis of IgG4-RD could not be assessed.

Mean serum IgG4 value was found significantly more important in the IgG4-RD patients. However, IgG4 values observed were overlapping with values observed in some other groups (Figure 1). 

As already reported, IgG4 elevation was found to be associated with cystic fibrosis [38], vasculitis [11, 14], and cancer [15]. More surprisingly, it was also associated with auto-immune diseases and patients with repeated infections or primary-immune deficiencies. We also show that IgG4 elevation can be associated to hypogammaglobulinemia in few patients with other IgG subclass deficiencies.

Cystic fibrosis has already been associated with serum IgG4 elevation during colonization and infection by *Pseudomonas aeruginosa* (present in our two patients) [42] and immediate-type hypersensitivity manifestations [38]. These observations, taken together with the observation of IgG4 elevation in a group of patients presenting with repeated infections, raise the question of the role of chronic infectious stimulation in IgG4 elevation. Cancer was associated with serum IgG4 elevation in 6.7% of patients in our study. Of note, a recent work found a more higher standardized incidence ratio for malignancies in IgG4-RD than in the general population [43]. However, none of our patients with cancer presented either clinical or histological evidence for IgG4-RD.

In 3 cases, a diagnosis of idiopathic interstitial lung disease was retained. Different intrathoracic involvements have been reported during IgG4-RD including interstitial lung disease [44]. Because of the absence of histological documentation obtained in these three patients, we cannot exclude an IgG4-RD with isolated or predominant lung involvement, especially in patient with highest serum IgG4 level.

Allergic manifestations were noted in only 10 patients (16.7%) in our study and could therefore not account for IgG4 elevation in this population. 

Our retrospective study clearly confirms that serum IgG4 elevation above 1.35 g/L is not specific for the diagnosis of IgG4-RD and can be observed in several clinical situations. The serum IgG4 elevation is more important in IgG4-RD but associated with an important variability within this group. Further studies are needed to define the sensibility and specificity of IgG4 values for the diagnosis of IgG4-RD. Until more specific biomarkers for IgG4-RD are made available, it must be kept in mind that several pathologies should be evoked end excluded in case of IgG4 elevation, before IgG4-RD diagnosis is retained. 

## 5. Conclusion

IgG4-related disease (IgG4-RD) is characterized by one or several fibroinflammatory organ involvements with typical pathological findings. A serum IgG4 elevation above 1.35 g/L is currently retained as an important biomarker of the disease, included in the diagnosis criteria. We confirm in this retrospective study, analyzing systematically the diagnosis associated with serum IgG4 above 1.35 g/L in a large unselected cohort of patients evaluated for IgG subclass, that IgG4 elevation is not specific of IgG4-RD. However, the patients with defined IgG4-RD presented with the most elevated serum IgG4 levels. Thus, several different pathologies should be excluded before IgG4-RD is retained in the context of serum IgG4 elevation.

## Figures and Tables

**Table 1:** Clinical reasons leading to IgG subclass measurement in patients with serum IgG4 elevation *n* = 59.

	% (*n*)
Hypogammaglobulinemia or PID suspicion	35.6% (21)
Hypergammaglobulinemia	25.4% (15)
IgG4-RD suspicion	23.7% (14)
Other	15.3% (9)

PID: primary immune deficiency; IgG4-RD: IgG4-related disease.

**Table 2:** Final diagnosis in patients with elevated serum IgG4 level (>1.35 g/L).

	*n* (%)	Mean IgG4 levels (g/L)
		(extremes)
Repeated infections	15 (25.4%)	2.31 (1.37–4.3)
Autoimmune diseases	8 (13.6%)	3.62 (1.38–11.3)
No final diagnosis	8 (13.6%)	1.94 (1.37–2.97)
IgG4-RD	6 (10.1%)	**12.64 (2.48−20.6)**
Possible IgG4-RD	5 (8.5%)	2.23 (1.56–3.37)
Cancer	4 (6.8%)	2.00 (1.71–2.32)
Primary immune deficiency	4 (6.8%)	1.80 (1.44–2.24)
Interstitial pneumonitis	3 (5%)	5.54 (1.51–12.7)
Cystic fibrosis	2 (3.4%)	4.49 (3.36–5.62)
Erdheim Chester disease	2 (3.4%)	3.05 (2.15–3.94)
Vasculitis	2 (3.4%)	3.68 (3.06–4.30)

IgG4-RD: IgG4-related disease.

Apart from the final diagnostic retained, allergic and atopic manifestations were found in 10 patients (16.9%).

**Table 3:** Pathologies (excepted IgG4-RD organ involvements) associated to serum IgG4 elevation in medical literature.

		Number of cases and references
Cancer	Pancreatic adenocarcinoma	13 cases [18], 5 cases [19], 1 case [20], 8 cases [16, 17], 2 cases [1], 11 cases [21], 1 case [22]
	Bile duct cancer/cholangiocarcinoma	3 cases [16, 17], 4 cases [22], 17 + 20 cases [23]
	Intraductal papillary mucinous neoplasm	1 case [16, 17]

Autoimmune diseases	Systemic lupus erythematosus	1 case [11], 2 cases [16, 17], 4 cases [our study], 1 case [24]
	Antiphospholipid syndrome	1 case [11], 2 cases [our study]
	Autoimmune hepatitis	1 case [16, 17]
	Rheumatoid arthritis	5 cases [11], 2 cases [25]
	Systemic sclerosis	3 cases [11], 1 case [our study]
	Sjögren's syndrome	3 cases [11], 2 patients [our study]
	Polymyositis/dermatomyositis	1 case [11]

ANCA-related vasculitis	Churg-Strauss syndrome*	4 cases [11], 4 cases [14]
	Microscopic polyangiitis	1 case [11], 1 case [our study]
	Nonspecified	1 case [16, 17]

Infections	Parasitic infections	2 cases [our study], specific IgG4 antibody elevation [3, 26–29]
	Bacterial infections	10 cases [our study]
	Viral infections	3 cases [our study]

Others	Multicentric Castleman's disease*	7 cases [11], 1 case [30], 1 case [16], 1 case [31], 5 cases [12]
	Eosinophilic disorders (fasciitis, pneumonia, and hypereosinophilic syndrome)	1 case each (fasciitis and pneumonia) [11], 2 cases of hypereosinophilic syndrome [16, 17]
	Chronic hepatitis	1 case [11], 2 cases (auto-immune) [32]
	Liver cirrhosis	2 cases [11], frequent cause of serum IgG4 elevation [33]
	Bronchial asthma	1 case [11], 3 cases [34]
	Idiopathic pulmonary fibrosis/interstitial pneumonia	1 case [11], 4 cases [16, 17], 3 cases [our study]
	Primary sclerosing cholangitis	1 case [16, 17], 3 cases [35], 12 cases [36], 33 cases [37]
	Chronic and idiopathic/acute pancreatitis	1 and 2 cases, respectively [16, 17], 4 and 5 cases, respectively [18]
	Behcet's disease	1 case [16, 17]
	Cystic fibrosis	7 cases [38], 2 cases [our study]

Pathologies in bold with * represent pathologies with especially frequent and high serum IgG4 elevation.
